# Homozygous Pathogenic Variant in Elongation Factor‐Like 1 (*EFL1*) as a Causal Factor in Shwachman‐Diamond Syndrome 2 in a Palestinian Child, With Distinct Ocular Manifestations

**DOI:** 10.1002/mgg3.70226

**Published:** 2026-04-27

**Authors:** Ibrahim Taha, Antonella Minelli, Cesare Danesino, Abdelrahman Swalmeh, Khalil Huraibat, Liana Al‐Labadi, Rema Obar, Khaled R. Beshtawi, Lubna Abu Samrah, Yousef Awlad Mohammad, Aya Farah, Abdulrahman Obar, Mutasem Khalaf, Orwa Nasser

**Affiliations:** ^1^ Optometry Department Arab American University Ramallah Palestine; ^2^ Department of Molecular Medicine University of Pavia Pavia Italy; ^3^ Paediatric Intensive Care, H. Clinic Ramallah Palestine; ^4^ Department of Oral and Maxillofacial Surgery, Oral Medicine, and Periodontology, Faculty of Dentistry University of Petra Amman Jordan; ^5^ Department of Audiology and Speech Therapy Birzeit University Birzeit Palestine; ^6^ College of Optometry University of Houston Houston Texas USA

**Keywords:** EFL1 mutation, endocrine dysfunction, ocular manifestations, Shwachman‐Diamond syndrome type 2, whole exome sequencing

## Abstract

**Background:**

Shwachman‐Diamond syndrome type 2 (SDS2) is a rare ribosomopathy caused by biallelic mutations in the *EFL1* gene. This condition presents with features similar to classic SDS1 such as pancreatic insufficiency and haematologic abnormalities.

**Case Presentation:**

We report a Palestinian female infant admitted to the NICU at H‐Clinic Hospital, Ramallah, in January 2023, with a homozygous mutation in the *EFL1* gene (c.3284G>A; p.Arg1095Gln), identified through whole exome sequencing and confirmed by Sanger sequencing. In addition to typical SDS2 features—pancytopenia, pancreatic insufficiency and growth failure—the patient exhibited unique manifestations, including ROP‐like retinal changes, infantile esotropia with inferior oblique overaction, and elevated ACTH and 17‐hydroxyprogesterone levels, indicating adrenal dysfunction.

**Findings:**

These findings expand the SDS2 phenotype to include novel ocular and endocrine involvement. No other pathogenic variants were identified by bioinformatic analysis. The recurrence of this variant in unrelated Palestinian families suggests a possible founder effect.

**Conclusion:**

This case underscores the importance of extended genetic testing and multidisciplinary evaluation in rare syndromes. It highlights the need for broader awareness of SDS2 among endocrinologists and ophthalmologists due to the novel clinical manifestations observed.

## Graphical abstract

We report a Palestinian female infant with a homozygous pathogenic *EFL1* variant (c.3284G>A; p.Arg1095Gln) causing Shwachman–Diamond syndrome type 2 (SDS2). Beyond the classical features of pancytopenia, exocrine pancreatic insufficiency, and growth failure, the patient showed previously unreported ocular manifestations—stage 2–3 retinopathy of prematurity, infantile esotropia with bilateral inferior oblique overaction, and anisometropic amblyopia risk—together with novel endocrine involvement (markedly elevated ACTH and 17‐hydroxyprogesterone). The recurrence of p.Arg1095Gln in unrelated Palestinian families suggests a possible founder effect. These findings expand the SDS2 phenotype and highlight the value of whole‐exome sequencing combined with multidisciplinary ophthalmological and endocrinological surveillance in infants with suspected ribosomopathies.

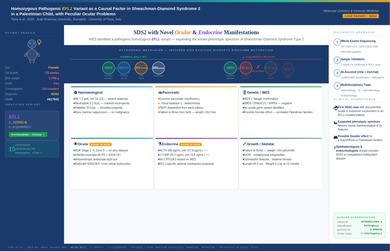

## 1 Introduction

Shwachman‐Diamond Syndrome (SDS) (OMIM 260400, SDS1 and OMIM 617941, SDS2) is a systemic disorder characterised by a constellation of symptoms, including neutropenia, pancreatic exocrine insufficiency leading to steatorrhoea and growth retardation. The patients usually present with short stature, skeletal dysplasia, and an elevated risk for bone marrow failure and leukemic transformation. These clinical features support a diagnosis of SDS. Additional complications, such as hepatic, cardiac, endocrine, behavioural, cognitive, ocular, dental and dermal abnormalities, are frequently observed, underscoring the syndrome's extensive systemic impact (Aggett et al. 1980; Bogusz‐Wójcik et al. 2021; Ginzberg et al. 1999; Taha et al. 2022; Trognon et al. 2024). Biallelic mutations in the *SBDS* gene, located at chromosome 7q11.21, are identifiable in approximately 90% of the clinically diagnosed cases (Boocock et al. 2003). Recent papers demonstrated genetic heterogeneity in SDS, with biallelic mutations in *DNAJC21* (Dhanraj et al. 2017) and *EFL1* (Stepensky et al. 2017; Tan et al. 2018, 2019) and heterozygous mutations in *SRP54* (Carapito et al. 2017). These mutations are associated with SDS or SDS‐like phenotypes, revealing a broader phenotypic spectrum.

SDS does not demonstrate any relevant ethnic predilection, presents with a slight male predominance, and its incidence is reported to range from 1/72,000 to 1/168,000 (Dror and Freedman 2002; Ginzberg et al. 1999; Minelli et al. 2012). The median onset age is 1.5 months (Hashmi et al. 2011), with diagnosis typically confirmed by 2 years. The mortality rate, particularly notable in early childhood and after the age of 30, underscores the relevance of early diagnosis and consequent management (Donadieu et al. 2012).

Clinical variability within SDS is profound; its features, such as neutropenia, developmental delays, pancreatic disorders and skeletal anomalies, vary significantly among individuals, even within the same family, making diagnosis difficult in some cases and highlighting the need for comprehensive genetic and clinical assessment (Burroughs et al. 2009; Myers et al. 2013; Thompson et al. 2022).

At the cellular level, cooperative action between *SBDS* and GTPase *EFL1* plays a critical role in ribosome assembly by catalyzing the detachment of eIF6 from the nascent 60S ribosomal subunit. The molecular basis of this interaction was established in foundational studies showing that *SBDS* acts as an allosteric regulator that cooperates with *EFL1* to catalyze eIF6 release, enabling 40S–60S subunit joining and formation of the functional 80S ribosome (Menne et al. 2007; Finch et al. 2011; Weis et al. 2015). Mutations in *EFL1* were associated with symptoms characteristic of SDS including infantile pancytopenia, pancreatic insufficiency and skeletal deformities (Stepensky et al. 2017; Tan et al. 2018, 2019). This observation allows classification of patients mutated in *EFL1* as SDS2 (Stepensky et al. 2017).

Morini et al. (2019) and Taha et al. (2022) used whole‐exome sequencing to underscore that extended genetic screening will lead to the comprehension of more complex phenotypes by identifying mutations additional to those causing the syndrome (Morini et al. 2019; Taha et al. 2022). In this case report, we present a case of SDS2 in a Palestinian infant, highlighting her distinctive phenotypic features.

## 2 Case Presentation

### 2.1 Patient Background

The patient is a Palestinian female infant, born prematurely in January 2023 at nearly 33 weeks of gestation to consanguineous parents who are first cousins. Following her delivery at H‐Clinic Hospital in Ramallah City, Palestine, she was admitted to the neonatal intensive care unit (NICU) due to intrauterine growth retardation (IUGR), and diagnosed as ‘newborn affected by slow intrauterine growth’ (Principal Diagnosis Code P05.9).

The patient presented with low birth weight (1740 g) and signs of pancreatic dysfunction as steatorrhoea. Additional clinical features observed included imperforate hymen, eczema, leukocoria and neonatal jaundice. Notable craniofacial dysmorphisms such as hypotelorism, wide nasal bridge and frontal bossing were also recorded.

Laboratory investigations revealed pancytopenia, hypocalcemia, elevated creatinine levels, hyperkalemia and hyponatremia. Exocrine pancreatic insufficiency was confirmed by low faecal elastase‐1 levels. At the age of 8 months the patient underwent surgical repair for bilateral inguinal hernias.

The patient's growth and development were closely monitored during her hospital stay, with continuous and comprehensive medical and laboratory evaluation aimed at identifying the cause of her haematological abnormalities and exocrine pancreatic dysfunction.

### 2.2 Haematological Findings

#### 2.2.1 Red Blood Cells (RBCs)

The red cells were normocytic and normochromic, with a few instances of anisopoikilocytosis. Crenated cells and fragmented cells were present in moderate or small numbers, respectively. Polychromasia appeared relatively normal, with no signs of rouleaux formation or RBC agglutination. Total haemoglobin (Hb) was 7.2 g/dL (neonatal reference range: 14–22 g/dL), and RBC count was 2.24 million/μL (reference: 4.1–6.1 million/μL). MCV was 96.3 fL, within the normal neonatal range (95–125 fL), indicating normocytic red cells.

#### 2.2.2 White Blood Cells (WBCs)

Total WBC count was 3.35 K/μL (reference: 9–30 K/μL), markedly below the normal neonatal range. Neutrophils were significantly reduced at 0.2 K/μL (reference: 1.5–8.5 K/μL), and lymphocytes were also reduced at 3.3 K/μL (reference: 4–13.5 K/μL), indicating leukopenia with marked neutropenia and relative lymphopenia, suggestive of bone marrow suppression. Monocytes, eosinophils, and basophils were within normal range.

#### 2.2.3 Platelets (PLTs)

Platelet count was markedly reduced at 35 K/μL (reference: 150–450 K/μL), indicating severe thrombocytopenia. MPV was elevated at 13.0 fL (reference: 7.0–11.0 fL), and PDW was markedly elevated at 59.1% (reference: 9%–17%), suggesting increased platelet turnover and a heterogeneous platelet population. No platelet clumping or activation was observed. Overall, the haematological profile indicated pancytopenia with no immature or malignant cells Table 1.

**TABLE 1:** Summary of key laboratory findings at presentation.

Parameter	Patient value	Reference range (neonatal)	Interpretation
Haemoglobin (Hb)	7.2 g/dL	14–22 g/dL	Severely reduced (anaemia)
RBC count	2.24 million/μL	4.1–6.1 million/μL	Markedly reduced
MCV	96.3 fL	95–125 fL	Normal (normocytic)
Total WBC count	3.35 K/μL	9–30 K/μL	Markedly reduced (leukopenia)
Neutrophils	0.2 K/μL	1.5–8.5 K/μL	Severely reduced (neutropenia)
Lymphocytes	3.3 K/μL	4–13.5 K/μL	Reduced (relative lymphopenia)
Platelets (PLT)	35 K/μL	150–450 K/μL	Severely reduced (thrombocytopenia)
MPV	13.0 fL	7.0–11.0 fL	Elevated; increased platelet turnover
PDW	59.1%	9%–17%	Markedly elevated; heterogeneous platelets
Faecal elastase‐1	Markedly low	> 200 μg/g	Exocrine pancreatic insufficiency
17‐OH progesterone	25.3 ng/mL	0.2–0.8 ng/mL	Markedly elevated; adrenal enzyme dysfunction
ACTH	685 pg/mL	8.3–57.8 pg/mL	Markedly elevated; primary adrenal insufficiency pattern
Serum calcium	Low	8.4–10.2 mg/dL	Hypocalcemia
Serum creatinine	Elevated	0.2–0.4 mg/dL	Elevated
Serum potassium	High	3.5–5.5 mEq/L	Hyperkalemia
Serum sodium	Low	135–145 mEq/L	Hyponatremia
TORCH serology (CMV, rubella, toxoplasma IgM)	All negative	Negative	Congenital infections excluded

### 2.3 Endocrine Function, Genetic and Immunological Status

A series of specialised tests were conducted to assess her endocrine, genetic and immunological status.

The 17‐hydroxyprogesterone (17‐OH progesterone) was measured based on a clinical suspicion of congenital adrenal hyperplasia (CAH), supported by signs suggestive of endocrine dysfunction including persistent electrolyte abnormalities (hyponatremia and hyperkalemia), unexplained jaundice, and failure to thrive.

17‐OH progesterone levels were markedly elevated, 25.3 ng/mL, significantly above the reference range for her age (0.2–0.8 ng/mL). This finding strongly suggests an adrenal enzyme deficiency, warranting further endocrine evaluation for possible adrenal gland disorders, particularly 21‐hydroxylase deficiency, the most common form of CAH.

The adrenocorticotropic hormone (ACTH) level was markedly elevated, 685 pg/mL (reference range: 8.3–57.8 pg/mL), and confirmed by two independent assay methods. Such a significant elevation suggests primary adrenal insufficiency and aligns with findings of elevated 17‐hydroxyprogesterone, supporting a possible diagnosis of congenital adrenal hyperplasia (CAH). This prompted consideration of further pituitary hormone assessment to evaluate the integrity of the hypothalamic–pituitary–adrenal (HPA) axis.

Human leukocyte antigen (HLA) typing revealed the presence of Class I alleles A02, A23, B13, B40, Cw04 and Cw06, as well as Class II alleles DRB113 and DRB108. These findings are relevant in evaluating potential immune‐mediated or genetic susceptibility to certain autoimmune or endocrine disorders, and may aid in future diagnostic or donor‐matching considerations.

Comprehensive immunological testing for congenital infections showed negative results for rubella IgM, *Toxoplasma gondii* IgM and cytomegalovirus (CMV) IgM, effectively ruling out these TORCH pathogens as contributors to the patient's clinical presentation.

### 2.4 Ocular Examination

As part of routine ophthalmological surveillance during the NICU admission, the patient underwent a comprehensive ophthalmological evaluation by a paediatric ophthalmologist. Fundoscopic examination revealed abnormal retinal features in the right eye consistent with stage 2–3 retinopathy of prematurity (ROP), located in posterior zone II and without plus disease (i.e., no posterior vessel dilation or tortuosity). Although the infant was born at nearly 33 weeks of gestation with a birth weight of 1740 g—above the threshold typically associated with severe ROP—her intrauterine growth restriction (IUGR) and prolonged multisystem complications may have contributed to her susceptibility to retinal vascular abnormalities. The left eye showed a clear red reflex with no retinal pathology. Both lenses were transparent, and anterior segment examination revealed no anomalies.

The first ophthalmic examination was performed at 4 weeks of age, in accordance with standard ROP screening guidelines for preterm infants with systemic risk factors. A biweekly retinal follow‐up schedule was implemented to monitor for ROP progression or regression.

She was also diagnosed with infantile esotropia of 60 prism diopters (PD) with inferior oblique overaction in both eyes (IOOA OU). Fixation behaviour was unsteady and poorly maintained, raising concern for developing amblyopia.

Cycloplegic refraction performed at 10 months of age revealed moderate hyperopia in the right eye (+4.50 diopters) and mild hyperopia in the left eye (+1.75 diopters), placing her at risk for anisometropic amblyopia of the right eye. Full‐time hyperopic spectacle correction was prescribed, and occlusion therapy was initiated with patching of the left (dominant) eye to encourage fixation in the amblyopic right eye.

Retinal function assessment was performed using the handheld RetEval LKC device, which enables non‐contact, portable electroretinographic and visual evoked potential (VEP) recording in non‐sedated infants. The ERG findings demonstrated a reduced b‐wave amplitude under photopic conditions, suggestive of inner retinal dysfunction, with relative preservation of the a‐wave, consistent with an inner retinal rather than photoreceptor‐level deficit at this stage. VEP responses showed prolonged P100 latency in the right eye compared to the left, consistent with delayed visual cortical maturation likely attributable to form deprivation and anisometropic amblyopia. These electrophysiological findings, in combination with the structural retinal changes, support the need for long‐term retinal surveillance given recent reports linking SDS2 with progressive rod‐cone dystrophies.

This constellation of findings—ROP stage 2–3, infantile esotropia with bilateral IOOA, and anisometropic amblyopia risk—represents a previously unreported ophthalmological manifestation in SDS2, underscoring the importance of early and sustained multidisciplinary ocular surveillance in affected infants.

## 3 Methods

### 3.1 Whole Exome Sequencing and Sanger Validation

Whole exome sequencing (WES) was performed on a peripheral blood sample using the IDT (xGen Exome Research Panel v2.0 combined with xGen Human mtDNA Research Panel v1.0) capture on an Illumina NOVASEQ 6000 platform. The FASTQ data generated were analysed using the Geneyx software with the DRAGEN pipeline, with the reference human genome (hg19). Sanger sequencing was employed to confirm and validate the identified genetic variant. WES was additionally performed on samples from both parents to determine carrier status and confirm the inheritance pattern of the identified variant; however, the parents declined this testing.

### 3.2 Bioinformatic Analysis

The variants were examined based on their position, gnomAD frequency, predicted mutation effect, inheritance pattern and data from mutation databases including ClinVar, LOVD and HGMD, as well as phenotype correlation through MalaCard, OMIM and HPO. Variant annotation and classification were performed using the eVai platform (enGenome Variant Interpreter), which integrates ACMG/AMP guidelines with phenotype‐driven algorithms for variant prioritisation. Functional impact was further evaluated using multiple in silico prediction tools including SIFT, REVEL, DANN, M‐CAP, FATHMM and PROVEAN, all of which consistently indicated a deleterious effect for the identified variant. Additionally, VarChat (https://varchat.engenome.com), developed by enGenome srl, was used to retrieve and summarise literature relevant to genomic variants using generative AI, enhancing the clinical interpretation process (De Paoli et al. 2024). Databases such as Franklin Genoox (https://franklin.genoox.com) and VarSome (https://varsome.com/) were also employed to cross‐validate variant classification and assess pathogenicity.

## 4 Result

### 4.1 Whole Exome Sequencing, Sanger Validation and Bioinformatic Analysis

WES identified a homozygous pathogenic variant in the *EFL1* gene: c.3284G>A (p.Arg1095Gln), rs376095522, located on chromosome 15 at position 82,422,793. This variant was confirmed by Sanger sequencing. In total, WES detected several variants; however, only this *EFL1* variant was interpreted as pathogenic and relevant to the patient's clinical phenotype. Importantly, no pathogenic or likely pathogenic variants were identified in other genes known to be associated with SDS including *SBDS*, *DNAJC21* and *SRP54*. Crucially, no pathogenic or clinically significant variants were identified in genes associated with ocular disease, retinal dystrophies, or other phenotypic features observed in this patient, further supporting the conclusion that the *EFL1* variant is the primary and sole genetic cause of the observed clinical presentation. Further analysis using the eVai platform confirmed the absence of any additional variants of pathogenic or uncertain clinical significance relevant to the patient's phenotype.

The identified *EFL1* variant (c.3284G>A, p.Arg1095Gln) has a very low allele frequency in the general population, reported at 0.000028 in the gnomAD database. According to ACMG guidelines, this variant is classified as pathogenic. In silico analysis using SIFT, REVEL, DANN, M‐CAP, FATHMM and PROVEAN consistently predicted a deleterious effect on protein function, further supported by VarSome and Franklin Genoox. The variant is listed in ClinVar (VCV000522584.3) as pathogenic, with clinical significance related to ribosome biogenesis disorders (last evaluated November 14, 2023). Parental genetic testing was recommended to determine carrier status and confirm the autosomal recessive inheritance pattern; however, the parents declined.

### 4.2 Clinical Implications

Mutations in the *EFL1* gene are associated with Shwachman‐Diamond syndrome type 2 (SDS2) (OMIM #617941), a rare ribosomopathy characterised by exocrine pancreatic insufficiency, haematopoietic abnormalities, short stature and metaphyseal dysplasia. The variant identified in our patient (rs376095522) has been previously reported in individuals presenting with a similar multisystem disease profile (Stepensky et al. 2017; Tan et al. 2018), further supporting its pathogenicity and clinical relevance.

At the latest follow‐up visit at age 12 months, the patient demonstrated persistent failure to thrive, with weight and length below the 3rd percentile for age (weight 6.1 kg, length 65.5 cm). She remained dependent on pancreatic enzyme replacement therapy, and faecal elastase‐1 levels continued to be significantly reduced, confirming ongoing exocrine pancreatic insufficiency. Ophthalmological reassessment confirmed persistent infantile esotropia with inferior oblique overaction in both eyes; ROP‐related retinal changes in the right eye remained stable without progression. Her haematologic profile continued to show mild pancytopenia, requiring close monitoring but not transfusion. Skeletal survey showed subtle metaphyseal irregularities, particularly in the femur and tibia, consistent with evolving metaphyseal dysplasia. Collectively, these findings support a diagnosis of SDS2 due to a homozygous *EFL1* variant.

Table 2 provides a structured chronological overview of the key clinical events, diagnostic findings and interventions from birth through the 12‐month follow‐up visit, illustrating the progressive multisystem complexity of this case.

**TABLE 2:** Timeline of key clinical events.

Time point	Clinical event/finding	Intervention/outcome
Birth, January 2023 (~33 weeks GA)	Premature delivery at H‐Clinic Hospital, Ramallah; IUGR; low birth weight (1740 g); NICU admission	Supportive NICU care initiated
Neonatal period (Weeks 1–4)	Pancytopenia (Hb 7.2 g/dL, WBC 3.35 K/μL, PLT 35 K/μL); leukocoria; neonatal jaundice; steatorrhoea; imperforate hymen; eczema; craniofacial dysmorphism (hypotelorism, wide nasal bridge, frontal bossing)	Haematological work‐up; nutritional support; ophthalmology referral
Neonatal period (Weeks 2–4)	Low faecal elastase‐1 (exocrine pancreatic insufficiency confirmed); elevated 17‐OH progesterone (25.3 ng/mL); elevated ACTH (685 pg/mL); HLA typing; TORCH serology (all negative)	Pancreatic enzyme replacement therapy initiated; endocrinology referral
Months 1–2	Stage 2–3 ROP right eye (posterior zone II, no plus disease); right infantile esotropia (~60 PD); inferior oblique overaction; right eye fixation unsteady	Biweekly retinal follow‐up scheduled; patching therapy planned
Age 8 months	Bilateral inguinal hernias diagnosed	Surgical repair performed
Age 10 months	Cycloplegic refraction: +4.50 D (RE), +1.75 D (LE); anisometropic risk identified	Full‐time hyperopic spectacle correction; alternate‐day patching initiated
Age 10–11 months	WES: homozygous EFL1 variant c.3284G>A (p.Arg1095Gln) identified; Sanger sequencing confirmation	Genetic counselling; parental testing recommended (declined by parents)
Age 12 months (last follow‐up)	Failure to thrive (< 3rd percentile); weight 6.1 kg, length 65.5 cm; stable mild pancytopenia; stable ROP; persistent esotropia; subtle metaphyseal irregularities (femur/tibia)	Continued PERT; multidisciplinary follow‐up (gastroenterology, haematology, ophthalmology, genetics)

## 5 Discussion

WES plays a pivotal role in the diagnosis and study of SDS and other rare genetic disorders by enabling precise identification of pathogenic mutations responsible for these conditions (Taha et al. 2022; Brlek et al. 2024; Glotov et al. 2023). Using WES and Sanger sequencing, we identified and validated in our patient the variant c.3284G>A (p.Arg1095Gln) in the *EFL1* gene, associated with SDS2. In the final step of ribosome maturation, the nascent 40S and 60S subunits are exported from the nucleus to the cytoplasm. Eukaryotic initiation factor 6 (eIF6) binds the 60S subunit within the nucleus to prevent premature association of the ribosomal subunits. The release of eIF6 in the cytoplasm requires the cooperative interaction of *EFL1* and *SBDS* proteins, acting as an allosteric switch that drives eIF6 eviction and enables formation of the functional 80S ribosome (Menne et al. 2007; Finch et al. 2011; Jaako et al. 2022).

The c.3284G>A (p.Arg1095Gln) variant has been identified in three affected siblings and an unrelated affected girl from Palestinian families diagnosed with SDS2 (Stepensky et al. 2017; Tan et al. 2018). Using *Saccharomyces cerevisiae* as a model organism, Stepensky et al. (2017) introduced the corresponding mutation and demonstrated that the mutant allele failed to complement a yeast *efl1Δ* strain, confirming its loss‐of‐function nature. The direct functional consequence—impaired eIF6 eviction from the 60S subunit by mutant *EFL1* proteins including p.Arg1095Gln—was subsequently demonstrated by Tan et al. (2019), who established this as the core pathogenic mechanism in SDS2 (Tan et al. 2019). Separately, molecular dynamics simulations on *EFL1* mutants, including p.Arg1095Gln, showed a peculiar rotation of domain IV with respect to domains I and II, resulting in an altered conformation compared to the wild‐type protein, suggesting an allosteric mechanism involving the concerted action of the GTPase domain and domain IV (Delre et al. 2020; Pérez‐Juárez et al. 2022). This variant is listed in ClinVar as pathogenic and associated with SDS2 (Variation ID: 522584, last evaluated November 14, 2023).

Kawashima et al. (2023) and Cario et al. (2024) provide summaries of previously reported SDS2 cases. We compare our patient with published cases, considering separately those sharing the same genotype and ethnic origin, and those with different biallelic mutations in *EFL1* and different ethnic origin (see Table 3).

**TABLE 3:** Genotype–phenotype comparison of reported SDS2 (*EFL1*) cases.

Source	Ethnicity	EFL1 variant(s)	Pancreatic insufficiency	Haematologic abnormality	Ocular findings	Endocrine findings
Current patient	Palestinian	Hom. p.Arg1095Gln	Yes (steatorrhoea, ↓faecal elastase‐1)	Pancytopenia, severe neutropenia	Leukocoria, ROP stage 2–3, esotropia + IOOA	↑17‐OHP, ↑ACTH (novel)
Stepensky et al. (2017) (4 patients, families B & C)	Palestinian	Hom. p.Arg1095Gln Hom. p.Met882Lys	Yes (diarrhoea, steatorrhoea)	Pancytopenia, severe neutropenia; transfusions required	None reported	None reported
Tan et al. (2018)	Not palestinian	Hom. p.Thr127Ala	Yes	Mild haematologic abnormalities	Deep‐set eyes; high myopia	None reported
Tan et al. (2019) (3 patients, France)	European	Compound het.: Arg754Ter/Phe505Ser; Cys883Gly + noncoding	Yes	BM failure, cytopenia	None reported	None reported
Lee et al. (2021) (3 Korean patients)	Korean	Compound het Thr1069Ala + other	Yes	Pancytopenia; somatic UPD mitigated severity	None reported	None reported
Cario et al. (2024)	Not specified	Compound het His30Arg/Asn867Asp	Yes (exo + endo)	Pancytopenia	None reported	Endocrine pancreatic insufficiency

### 5.1 Comparison With Palestinian Cases (Same Genotype)

The clinical comparison between our patient and those described by Stepensky et al. (2017) with the same homozygous mutation (c.3284G>A, p.Arg1095Gln), in individuals B‐II‐1, B‐II‐3, B‐II‐5, C‐II‐1 from families B and C originating from two unrelated consanguineous Palestinian‐Muslim families, highlights shared haematological abnormalities, growth failure and pancreatic insufficiency. Our patient resembles those reported by Stepensky et al. in presenting with low birth weight and intrauterine growth restriction (IUGR). At 12 months of age, she exhibited failure to thrive, with weight at 6.1 kg (< 3rd percentile) and length at 65.5 cm (< 3rd percentile), although her growth impairment appeared less severe compared to the Stepensky cohort. Pancreatic insufficiency was a shared finding, but while those patients reported by Stepensky et al. had more pronounced gastrointestinal manifestations—including diarrhoea and steatorrhoea in 3 of the 4 untreated cases—our patient's symptoms were better controlled under enzyme replacement therapy.

Pancytopenia was observed in all cases; however, those patients reported by Stepensky et al. experienced more severe and symptomatic neutropenia and thrombocytopenia, requiring repeated blood transfusions. In contrast, our patient showed moderate cytopenia that has so far been managed conservatively. Skeletal anomalies were more pronounced in those patients reported by Stepensky et al., who exhibited rhizomelic limb shortening and metaphyseal dysplasia, whereas our patient presented with facial dysmorphic features—including hypotelorism and a broad nasal bridge—but showed no significant skeletal abnormalities upon clinical and radiological evaluation at 9 and 12 months of age.

Ocular manifestations were the main distinguishing feature: our patient presented with stage 2–3 ROP, infantile esotropia with bilateral inferior oblique overaction (IOOA OU), and anisometropic amblyopia risk, whereas those patients reported by Stepensky et al. had no documented ocular involvement. Both groups had additional complications; our patient experienced neonatal jaundice, eczema, endocrine dysfunction (elevated 17‐OH progesterone and ACTH), and underwent inguinal hernia surgery, whereas those reported by Stepensky et al. presented with microcephaly, muscle hypotonia and developmental delay. These differences collectively suggest a broader phenotypic spectrum potentially associated with this genotype.

An important interpretive consideration is distinguishing features attributable to the homozygous *EFL1* mutation from those that may be secondary to prematurity (~33 weeks gestation). Retinopathy of prematurity (ROP) is a recognised complication of preterm birth, particularly in neonates requiring prolonged oxygen supplementation in the NICU, and cannot be definitively attributed to the genetic disorder in isolation. However, several features in our patient are not readily explained by prematurity alone and are attributed, at least in part, to *EFL1* dysfunction: (1) exocrine pancreatic insufficiency confirmed by markedly reduced faecal elastase‐1—a hallmark of SDS not caused by prematurity; (2) severe pancytopenia with marked neutropenia, consistent with SDS‐associated bone marrow failure; (3) markedly elevated 17‐OH progesterone and ACTH, indicative of adrenal dysfunction beyond what would be expected from prematurity alone and (4) the same homozygous *EFL1* p.Arg1095Gln variant has been identified in other Palestinian patients who presented with similar core SDS2 features (Stepensky et al. 2017). The absence of parental genotyping is acknowledged as a limitation of this report. Future molecular studies, including functional assessment of patient‐derived cells, would strengthen the causal attribution.

Although our patient and those reported by Stepensky et al. (2017) share the same homozygous *EFL1* mutation (p.Arg1095Gln), there is no evidence of a familial or direct geographical relationship between them. Both patient groups come from consanguineous families, consistent with the high inbreeding coefficient among Palestinians. The families are from different cities approximately 60 km apart. The recurrence of the same mutation in unrelated Palestinian patients may suggest a founder effect, though further genealogical and population genetic studies are required to confirm this hypothesis.

### 5.2 Comparison With Additional SDS2 Cases

A 14‐year‐old girl described by Tan et al. (2018) with homozygous EFL1 Thr127Ala exhibited pancreatic insufficiency and haematological abnormalities and survived into adolescence, suggesting a potentially milder clinical progression. Unlike our patient, no ocular or endocrine complications were reported. Tan et al. (2019) described three additional patients with compound heterozygous and homozygous *EFL1* mutations, showing more pronounced skeletal deformities and developmental delays without ocular or endocrine involvement. Cario et al. (2024) reported a fatal SDS2 case in a female infant with compound heterozygous *EFL1* variants (His30Arg and Asn867Asp), with dominant pulmonary pathology and cardiopulmonary failure at 18 months—features not observed in our patient. Korean patients described by Lee et al. (2021) with compound heterozygous *EFL1* mutations experienced somatic uniparental disomy in bone marrow cells, which appeared to mitigate some catastrophic symptoms; none exhibited ocular complications. See Table 3.

### 5.3 Ocular Manifestations in SDS and SDS2

Studies have identified a range of ocular features in SDS patients including retinitis pigmentosa, coloboma, strabismus and punctate keratitis (Aggett et al. 1980; Ginzberg et al. 1999; Zhang et al. 2025). Case reports have documented rarer manifestations such as periorbital cellulitis, involvement of cilioretinal arteries, and ocular changes associated with myelodysplastic syndrome (Breazzano and Benegas 2017; Keogh et al. 2012). Importantly, individuals with SDS‐like phenotypes caused by mutations in *DNAJC21* and *EFL1* exhibited deep‐set eyes, retinal dystrophies, and significant visual impairments including high myopia (Dhanraj et al. 2017; Tan et al. 2018). Notably, Zhang et al. (2025) reported a 10‐year follow‐up of a patient with a clinical diagnosis of SDS and severe rod‐cone dystrophy, demonstrating progressive photoreceptor degeneration despite stable vision; no molecular genetic diagnosis was established in that case, leaving the underlying genetic aetiology undetermined. Recent studies have also highlighted inflammatory eye conditions in SDS patients such as bilateral blepharoconjunctivitis, episcleritis, keratoconjunctivitis, corneal neovascularisation and infiltrates (Furutani et al. 2020). Our patient uniquely presented with stage 2–3 ROP, infantile esotropia with bilateral inferior oblique overaction, and anisometropic amblyopia—a constellation of findings not previously documented in SDS2.

### 5.4 Endocrine Manifestations in SDS and SDS2

Endocrine dysfunctions are common in SDS, with approximately 63% of patients experiencing issues such as growth hormone deficiency, hypothyroidism, congenital hypopituitarism and type 1 diabetes mellitus (Bogusz‐Wójcik et al. 2021; Minelli et al. 2025). These patients also tend to have lower height and BMI compared to the general population. Additional features, such as elevated thyrotropin, abnormal glucose levels and elevated follicle‐stimulating hormone (FSH) following bone marrow transplantation, have been reported (Jivani et al. 2016; Myers et al. 2013).

Notably, endocrine dysfunction as documented in previously published SDS patients (predominantly *SBDS*‐mutated) has primarily involved the growth hormone axis and thyroid function, with ACTH levels typically within or below normal range (Bogusz‐Wójcik et al. 2021). In striking contrast, our patient—who represents the first documented *EFL1*‐mutated SDS2 case with endocrine involvement—presented with markedly elevated ACTH (685 pg/mL) and 17‐hydroxyprogesterone (25.3 ng/mL). The elevation of ACTH is consistent with a primary adrenal insufficiency pattern or adrenal hyperstimulation due to downstream enzyme dysfunction. The elevated 17‐OHP raises the possibility of 21‐hydroxylase pathway disruption as seen in congenital adrenal hyperplasia, though no pathogenic variant in *CYP21A2* was identified on WES. It is possible that this endocrine dysregulation represents a novel, *EFL1*‐specific pathophysiological mechanism, perhaps related to the global translational defect caused by impaired ribosome maturation affecting adrenal steroidogenic cells. This hypothesis warrants further investigation through adrenal stimulation testing and longitudinal hormonal monitoring in future SDS2 patients.

### 5.5 Significance of Extended Genetic Analysis

The identification of a new patient with a homozygous pathogenic variant in the *EFL1* gene as a causal factor in SDS2 further strengthens our understanding of the genetic heterogeneity of this condition. The role of *EFL1*, which accounts for only a minority of SDS‐like phenotypes, underscores the importance of extended genetic analysis—particularly WES—in uncovering the molecular basis of rare diseases. Our findings highlight the value of integrating advanced diagnostic tools, including whole exome sequencing and AI‐assisted variant interpretation, into clinical practice to support personalised medicine approaches in SDS and related phenotypes. These findings also emphasise the need for multidisciplinary clinical awareness: the diagnosis of SDS2 should be considered not only by haematologists and gastroenterologists, but also by ophthalmologists and endocrinologists, given the expanding phenotypic spectrum demonstrated by this case. Future research should aim to define the full spectrum of genetic mutations contributing to SDS and related disorders, characterise their functional consequences, and investigate possible founder effects in specific populations.

## Author Contributions

**Ibrahim Taha** conceptualized and coordinated the study, performed clinical ophthalmological evaluation and drafted the manuscript. **Antonella Minelli** and **Cesare Danesino** contributed to the genetic analysis and bioinformatic interpretation, manuscript writing and supervision. **Abdelrahman Swalmeh** and **Abdulrahman Obar** were responsible for NICU and paediatric evaluation. **Khalil Huraibat**, **Liana Al‐Labadi**, **Lubna Abu Samrah** and **Rema Obar** assisted in clinical documentation, lab data analysis and manuscript editing. **Khaled R. Beshtawi** provided radiological and developmental assessments. **Yousef Awlad Mohammad** and **Aya Farah** contributed to patient follow‐up, clinical data collection and manuscript preparation. **Mutasem Khalaf** contributed to audiological and speech‐language assessment and clinical documentation. **Orwa Nasser** contributed to study conceptualisation, clinical oversight, critical revision of the manuscript and supervision. All authors reviewed and approved the final manuscript.

## Funding

The authors have nothing to report.

## Ethics Statement

This study was conducted in accordance with the Declaration of Helsinki. As a single de‐identified case report involving routine clinical care, this study qualifies for exemption from full Institutional Review Board (IRB) review under the applicable Palestinian Ministry of Health guidelines governing human subjects research, which exempt retrospective case reports that do not involve experimental intervention, in accordance with internationally recognised criteria for case report ethics (CARE guidelines). No experimental procedures beyond standard clinical management were performed, and patient anonymity has been preserved throughout.

## Consent

Written informed consent for clinical examination, laboratory testing, genetic analysis and publication of anonymized clinical data, including clinical photographs and genetic data, were obtained from the patient's parents.

## Conflicts of Interest

The authors declare no conflicts of interest.

## Data Availability

The data that support the findings of this study are available on request from the corresponding author. The data are not publicly available due to privacy or ethical restrictions.
